# Recent advances in microsystem approaches for mechanical characterization of soft biological tissues

**DOI:** 10.1038/s41378-022-00412-z

**Published:** 2022-07-07

**Authors:** Enming Song, Ya Huang, Ningge Huang, Yongfeng Mei, Xinge Yu, John A. Rogers

**Affiliations:** 1 Shanghai Frontiers Science Research Base of Intelligent Optoelectronics and Perception, Institute of Optoelectronics, Fudan University, Shanghai, 200433 China; 2 International Institute of Intelligent Nanorobots and Nanosystems, Fudan University, Shanghai, 200433 China; 3 Department of Biomedical Engineering, City University of Hong Kong, Hong Kong, 999077 China; 4 Department of Materials Science, Fudan University, Shanghai, 200433 China; 5 Querrey Simpson Institute for Bioelectronics, Department of Materials Science and Engineering, Departments of Biomedical Engineering, Neurological Surgery, Chemistry, Mechanical Engineering, Electrical Engineering and Computer Science, Northwestern University, Evanston, IL 60208 USA

**Keywords:** Electrical and electronic engineering, Engineering

## Abstract

Microsystem technologies for evaluating the mechanical properties of soft biological tissues offer various capabilities relevant to medical research and clinical diagnosis of pathophysiologic conditions. Recent progress includes (1) the development of tissue-compliant designs that provide minimally invasive interfaces to soft, dynamic biological surfaces and (2) improvements in options for assessments of elastic moduli at spatial scales from cellular resolution to macroscopic areas and across depths from superficial levels to deep geometries. This review summarizes a collection of these technologies, with an emphasis on operational principles, fabrication methods, device designs, integration schemes, and measurement features. The core content begins with a discussion of platforms ranging from penetrating filamentary probes and shape-conformal sheets to stretchable arrays of ultrasonic transducers. Subsequent sections examine different techniques based on planar microelectromechanical system (MEMS) approaches for biocompatible interfaces to targets that span scales from individual cells to organs. One highlighted example includes miniature electromechanical devices that allow depth profiling of soft tissue biomechanics across a wide range of thicknesses. The clinical utility of these technologies is in monitoring changes in tissue properties and in targeting/identifying diseased tissues with distinct variations in modulus. The results suggest future opportunities in engineered systems for biomechanical sensing, spanning a broad scope of applications with relevance to many aspects of health care and biology research.

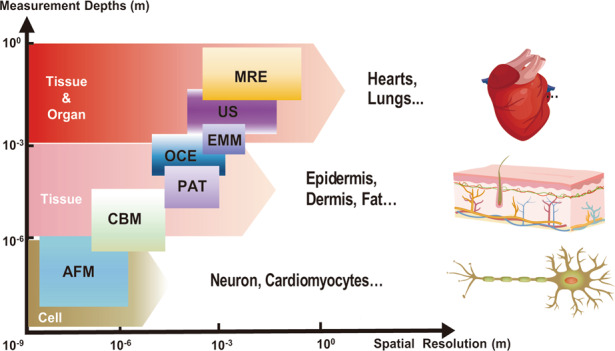

## Introduction

Emerging technologies that can quantify the mechanical properties of soft biological tissues in living organisms across a variety of spatial scales and resolution levels have several possible uses in biomedical research and clinical disease diagnostics^1–5^. Many of the most advanced approaches rely on microsystems technologies, with embodiments that range from miniaturized durometers for objective assessment of disease severity in venous edema^6,7^ to fine biopsy needles for biomechanically targeting cancerous or fibrotic tissues^8,9^. Particular interest is in the measurement of elastic moduli as the basis for clinical assessments of various conditions^3,10,11^ including dermatological pathologies, such as scleroderma and psoriasis^12–15^, and deep tumors associated with breast cancer and hepatocellular carcinoma^16–21^. Further diagnostic utility focuses on monitoring time-dependent changes in the elastic modulus of biomaterials and tissues associated with growth, aging, regeneration, and wound healing^22–24^.

Conventional methods for evaluating tissue moduli rely on bulk measurements of displacement as a function of applied forces, including those associated with torsion, compression, suction, and indentation, typically via the use of external equipment in an invasive manner^25–29^. Despite the value of these schemes for measuring skin mechanics^3,10^, the designs, integration approaches, and modes of interfacing such technologies with soft tissues limit relevant applications to episodic evaluations in hospital settings by trained personnel. Of note is optical coherence elastography (OCE) which combines light transmission in tissues with photophysical interactions to enable mechanical assessments of soft tissue properties^30^. Illumination of targeted tissue and recording of the reflected light attenuated by absorption and scattering within tissues can yield biometric information such as tissue stiffness and arterial blood pressure^31,32^, typically with spatial resolution in the submillimeter range and a penetration depth of many millimeters. The most recent alternative methods exploit advanced techniques of magnetic resonance elastography (MRE)^33^; this scheme is capable of spatial-temporal imaging at high spatial resolution (~1 mm), deep-tissue profiling (~1 m), and quantitative mapping of tissue stiffness based on the propagation of shear waves deep inside tissue/organ structures in a noninvasive manner^34^. These high-cost tools, however, require elaborate setups and hospital/laboratory practitioners, thereby precluding rapid, direct use for at-home diagnostic monitoring or continuous tracking.

Recently developed classes of electromechanical microsystems that incorporate ultrathin, flexible piezoelectric arrays of actuators/sensors are of growing interest in this context as conceptually distinct types of approaches for tissue modulus measurements in tissue-compatible formats^35,36^. Key distinguishing features include miniature dimensions, mechanically compliant architectures, and minimally invasive interfaces to complex textures of biological surfaces^37^, with precise measurements of minute deformations of soft tissues at near-surface depth and spatial resolution both at the microscale^38^. Examples include filamentary penetrating probes for in vivo mechanical sensing^39^, deformable, piezoresistive cantilevers for local cell/tissue biomechanics^40^, and conformal sheets for spatial mapping of skin moduli^41^. Although valuable in many scenarios, a limitation of these approaches is their lack of ability to sense deep-tissue profiles due primarily to their miniaturized device designs, limiting their use to superficial measurement depths (typically tens of microns)^30^.

**Fig. 1:**
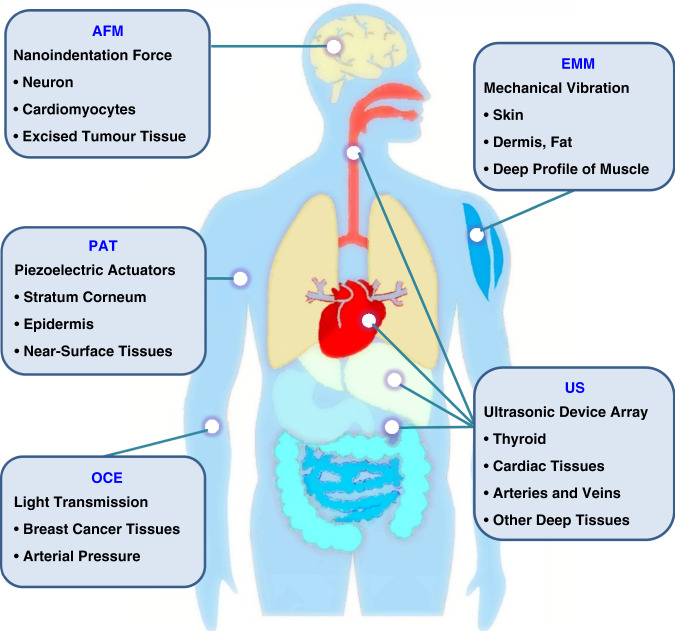
A summary graphical abstract for recently established microsystem technologies for biomechanical evaluations. Illustrations of each technology involve key features and corresponding biological measurement targets.

Recent research has established classes of miniature electromechanical modulus (EMM) sensors that combine a vibratory actuator with a soft strain-sensing sheet for real-time measurements of tissue biomechanics at characteristic depths of up to ~8 mm and a spatial resolution in the millimeter range^42^. Alternative platforms for depth profiling exploit wearable arrays of ultrasonic transducers via ultrasound (US), with additional capabilities in capturing blood pressure waveforms from deeply embedded vessels^43^. These ultrasound characteristics for deep-tissue sensing (>10 cm) at a spatial resolution of hundreds of micrometers can complement those of methods for biomechanical sensing at superficial depths. Figure 1 shows a summary of recently established microsystem technologies, each followed by key features and corresponding applicable biological targets, including standard atomic force microscopy (AFM)/microcantilever probes^44,45^, piezoelectric-actuator/sensor techniques (PAT)^41^, EMM sensors^42^, OCE methods^31^ and US schemes^43^. The overall need is in these and other complementary approaches that support simple, rapid but precise evaluation of soft tissue biomechanics across a variety of scales and depths, from thicknesses in the submicron cellular regime to large, deep-tissue geometries.

The following summarizes the latest progress in microsystem approaches that focus on characterization of the mechanical properties of various soft biological tissues, as presented in Fig. 1. The emphasis is on operational concepts, material selections, engineering designs, and system attributes of approaches that have some proven diagnostic utility in terms of different measurement scales. The article begins with an overview of various types of electromechanical devices in forms that range from flexible penetrating filaments to shape-conformal sheets and stretchable ultrasonic-transducer arrays. The following sections review the use of planar MEMS-based technologies for sensing across length scales that span single cells to complete organs in live animal models and the human body. One highlight includes miniature electromechanical devices for depth profiling of soft tissue biomechanics across tunable characteristic depths^42^. Comparative results define the advantages and disadvantages of these various methods. Applications include the monitoring of changes in tissue mechanical properties and targeting of abnormal regions associated with different diseases. Recent advances in these areas may define a future where tissue mechanics can form a prominent aspect of health care and biomedical research.

### Mechanical behaviors of soft biological tissues

**Fig. 2:**
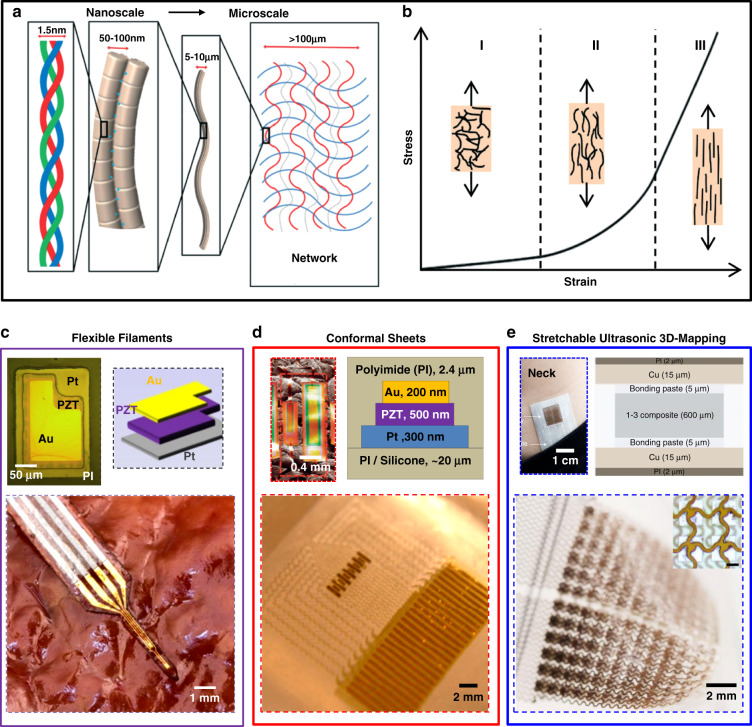
Emerging classes of flexible/stretchable microsystem platforms for evaluating the elastic moduli of soft tissues. **a** Schematic illustration of microstructures in soft biological tissues, with a focus on the skin. Reproduced with permission^47^. Copyright 2015, Nature Publishing Group. **b** The resultant mechanical properties exhibit “J-shaped” stress–strain behavior. Reproduced with permission^46^. Copyright 2017, Royal Society of Chemistry. **c**–**e** Various flexible/stretchable microsystem approaches, with upper insets showing the material engineering and manufacturing technologies. **c** Needle-shaped flexible probe for tissue modulus measurements based on an ultrathin actuator and sensor placed on biological tissue. Reproduced with permission^65^. Copyright 2018, Nature Publishing Group. **d** Photograph of a conformal device on a silicone sheet substrate for characterization of the elastic modulus of skin. Reproduced with permission^41^. Copyright 2015, Nature Publishing Group. **e** Optical image of a stretchable phased array consisting of 12 × 12 interconnected ultrasonic transducers during bending and stretching. Inset: the magnified image of four transducer units in the array, with a spacing pitch of ~0.8 mm. Scale bars, 300 µm (inset). Reproduced with permission^66^. Copyright 2021, Nature Publishing Group

Soft biological tissues in animal and human subjects, from the skin to various internal tissues, generally feature compliant mechanics and levels of stretchability that can accommodate deformations induced by natural motions or externally applied forces. The general nature of their biomechanics involves so-called “J-shape” stress–strain behaviors that typically include three different characteristic regions (Stages I–III) in the relationship between stress and strain^46–48^. These responses result from the constituent biomaterials and their wide-ranging levels of hierarchy (Fig. 2a), from nanoscale elements, such as the collagen triple helices, to submicron-scale composites, such as collagen fibrils and collagen fibers, to microscale networks, such as chained microstructures and resultant interconnected geometries. Such J-shaped mechanical behaviors apply across a range of biological systems, from cells to tissues and organs, including human ligaments^49^, blood vessels^50^, muscles^51^, brain tissues^45^, and even spider silk^52^. An exception is in fatty tissues, where the stress and strain are approximately linear due to material phases and viscoelastic effects^53^.

The following discussion uses skin as a typical example. Human and animal skin involves structures that span from nanoscale dimensions of twisted collagen triple helices (<100 nm diameter) to microscale networks of wavy collagen and elastin fibers (>100 µm dimensions, as shown from left to right in Fig. 2a), thereby leading to different mechanical properties under various corresponding physical conditions. Specifically, Fig. 2b demonstrates a representative J-shaped stress–strain curve for skin under stretching, with three different stages at increasing levels of stress and associated strain^46^. Stage I corresponds to the elastic-response region at small strains, where the compliant, wavy network of collagen fibers presents low stiffness and a linear relationship between stress and strain that defines the modulus. Here, the tissue can freely extend with minimal applied forces (that is, stress) and a small elastic modulus. With increasing strain and stress, the system enters Stage II, where mechanical deformation distributes across the entire wavy network, as each collagen fiber begins to uncoil. As such, the tissue stiffens, with a pronounced increase in the tissue modulus. As the strain increases further to Stage III, the collagen fiber network straightens and enters a state of tension, where the stress–strain behavior depends on the mechanical properties of the fibers themselves, as opposed to their geometrical configurations. As such, the results in this region again show a relatively linear response between stress and strain, but with a large tissue modulus until, at higher strains, mechanical failure occurs (after Stage III)^47^.

Although similar in the characteristic shapes of such stress–strain curves, the strain ranges, and associated modulus values vary largely from various tissues and different locations among animal and human subjects. As an example, uniaxial tensile measurements of abdominal skin in a human subject (10 cm × 0.5 cm) present typical J-shaped stress–strain curves, with a low strain range of 0–0.5 for Stage I, ~0.5–0.8 for Stage II and >0.8 for Stage III^54^, corresponding to modulus values of 0.3–0.7 MPa, 0.7-20 MPa and ~20 MPa for each regime (maximum tensile strength of ~10 MPa), respectively^55^. Another example is the tensile response of the skin from the backs of rabbit models (2 cm × 0.4 cm), with approximate strain ranges of ~0–0.5, ~0.5–1.0, and ~1.0–1.8 for Stage I, Stage II and Stage III, respectively, where the associated stress–strain slope increases monotonically with increasing strain and therefore achieves a maximum tensile strength of ~16 MPa before mechanical failure at a measurement strain rate of 10^−1^ s^−1^^47^.

Precise evaluation of these mechanical properties can facilitate assessments of various pathophysiologic conditions^56^. An important emphasis is on the elastic modulus evaluated in Stage I, as depicted in Fig. 2b. The results can serve as the basis for noninvasive measurements of tissue stiffness and their associated medical significance, such as those related to dermatological evaluations of skin disorders (e.g., scleroderma, psoriasis)^1^. Tissues of the human body exhibit a remarkable range of elastic modulus values from 1 kPa (brain) to a few GPa (bone)^1^. Among soft tissues, those of the brain have the lowest elastic moduli from 1–3 kPa^57^, followed by those of the liver (~6.5 kPa)^58^, kidney (4–8 kPa)^59^, and spleen (~20 kPa)^60^. High modulus tissues include ligaments (25–93 MPa)^61^ and bones. The mechanical properties also depend on conditions such as health status, age, and pathophysiology. For instance, inflammation and fibrosis can increase the modulus of the liver to >40 kPa for patients with life-threatening conditions compared to values for healthy individuals (~6.5 kPa)^62^. Malignant carcinoma can increase thyroid tissue stiffness from healthy values of 9–11.4 kPa to 44–110 kPa^1^. For the skin, age and hydration can alter the biomechanics^63^, where stiffer responses occur with increasing age and decreasing levels of hydration^64^. As an example, the modulus of the abdominal epidermis is >10 MPa among older subjects (age: 66–69) compared to values of 6 MPa for younger individuals (age: 33–35)^41^, mostly as a consequence of reduced hydration levels in the former.

### Flexible/stretchable microsystems

This section focuses on emerging microsystem technologies and material strategies for in vivo mechanical measurements of soft biological tissues that rely on minute deformations of tissues (Stage I) in deployable designs of clinical relevance and, in some cases, home use, where safety, reliability, and high measurement precision represent key considerations. In many cases, the engineering goals are in miniaturized dimensions, compliant designs and tissue-like mechanics matched to those of biological targets over brief time intervals or persistently interfaced. Here, a focus is on measurement platforms that offer mechanical compliance to the targeted tissues; some recent examples are shown in Fig. 2c–e, with architectures including (c) needle-shaped penetrating filamentary probes^65^, (d) thin, conformal electronic platforms on flexible sheets^41^ and (e) stretchable arrays of ultrasonic transducers for deep-tissue characterization^66^. These microsystems with tissue-like mechanics are distinct in their flexible/stretchable forms and their ability to offer stable measurements with minimally invasive interfaces to biological surfaces, as opposed to traditional, rigid devices that rely on bulk measurements of displacement as a function of large applied forces, with associated measurement uncertainties and difficulties in application to curved, time-dependent tissue surfaces.

In all cases, manufacturing techniques adapted from the integrated circuit industry can serve as an entire or partial basis for device fabrication. The filamentary tissue-penetrating microsystem for tissue modulus measurements^65^ referenced above is depicted in Fig. 2c. The probe incorporates a mechanical actuator and sensor constructed from ultrathin layers of the piezoelectric material lead zirconate titanate (PZT, 200 µm long, 140 µm wide, 500 nm thick) with metal interconnections (Au/Pt, 200/200 nm thick). For material engineering and manufacturing strategies, the PZT films exploit sol-gel techniques and patterning by photolithography and etching for connections to multilayer electrodes, subsequently transferred onto flexible polyimide (PI) substrates (75-µm thick) via transfer printing approaches, as shown in the upper inset. For measurement purposes, applying a sinusoidal voltage to the actuator induces small-scale mechanical deformations of the surrounding materials as well as adjacent contacting tissues with the same time dynamics, thus resulting in a detectable voltage at the driving frequency. The thin geometry and narrow width of the tip (4 mm long, 200 µm wide) achieved by laser ablation allows for delivery through soft tissues into targeted regions at a certain depth and for lamination onto standard medical needles for biopsies. In both cases, the measurements can distinguish abnormal tissues during the assessment of organ pathology through variations in tissue modulus. In vivo experiments on live rat models demonstrate successful characterization of the elastic moduli of different organs (i.e., liver, fat, spleen, etc.)^65^, consistent with ex vivo measurements performed after explantation. Ongoing efforts have been focused on clinical studies of guided mechanical targeting of diseased/abnormal tissues, with these microsystems as a complementary approach to other recent diagnostic methods such as those based on MRE.

Conformal arrays of actuators/sensors in sheet-like architectures yield flexible, surface-mounted systems capable of covering large areas in a nonpenetrating fashion, with the ability to follow complex textures of targeted tissue surfaces^41^. A representative example is shown in Fig. 2d. Here, the material engineering includes 7 actuators and 6 sensors (500 nm thick PZT materials) fabricated via sol-gel techniques with serpentine interconnections formed using photodefined metal traces (Au/Pt, 200/300 nm thick) encapsulated by layers of PI (2.4 µm thick). Subsequent transfer printing enables integration on an elastomeric sheet of silicone (20 µm thick) to support the device platform, thus facilitating direct noninvasive coupling to surfaces of the skin or other tissues, as shown in the upper inset of Fig. 2d. The results allow for the clinical characterization of soft tissue biomechanics, with operational principles similar to those of the devices in Fig. 2c. Evaluations performed at various locations across the human body yield data regarding the dependence of modulus on age, position, and skin preparation (e.g., application of moisturizer). The results reveal an increase in modulus with age and a temporary reduction (within 5 min) from ~6 MPa to ~3 MPa after applying moisturizers, consistent with expectations. Clinical studies with these same devices on patients with dermatological malignancies suggest potential utility in tracking the progression of skin cancers, as outlined in examples that follow.

Fully stretchable electronic systems in similar array-based, sheet-like designs are also of interest^66^, as shown in the ultrasonic device array for three-dimensional (3D) mapping of the physical properties of deep tissues in Fig. 2e. The array includes 12 × 12 ultrasonic transducers (a unit area of 550 × 550 µm^2^, a 600 µm thick, pitch spacing of 770 µm), where the transducers involve 1–3 piezoelectric composite elements formed via dice-and-fill techniques. Circuit patterns include serpentine interconnections of copper (Cu) traces embedded in a photodefined PI mesh (4 µm thick), fabricated by a laser ablation system, followed by transfer printing onto a stretchable substrate of silicone elastomer. Detailed information on the material engineering aspects is shown in the upper inset of Fig. 2e. As such, the device of this design shows an elastic response to tensile strains as large as 16% for stable ultrasonic monitoring across deformations characteristic of human skin. As follows, each transducer electromechanically couples to the skin in a way that efficiently converts electrical power to vibrational power^67^ via a phased-array control technique; this technique allows active focusing and steering of beams of ultrasound across a range of incident angles (−20° to 20°) into deep-tissue regions (~14 cm beneath the skin) with negligible degradation in signal quality (signal-to-noise ratio >18 dB). In vivo evaluations include real-time sensing of deep-tissue mechanical structures and continuous monitoring of tissue physical properties such as major arteries and veins^66^, with capabilities for measuring cerebral blood flow quantitatively in real-time. Such acoustic methods, including ultrasound and acoustic radiation force impulse imaging, generate propagating waves inside tissues as the foundation of measurements based on small-scale displacements^68^. Given the density and thickness of tissue layers, ultrasound waves propagate at velocities that can be determined from pulse echo durations recorded via an ultrasonic transducer. The velocity correlates with tissue physical properties (e.g., density, modulus) and can be used to determine tissue biomechanics, such as Young’s modulus^69^. Current work focuses on miniaturization, enhanced spatial resolution, and integration with wireless data transmission capabilities and battery-free designs as skin-mounted platforms for wearable diagnostics.

In addition to direct measurements of tissue elastic moduli, recent research also includes other types of mechanical assessments relevant to strain-sensing, such as strain distribution and mapping via active semiconductor nanomembrane device arrays, where silicon nanomembranes (Si-NMs) serve as piezoresistive elements with high-sensitivity^70^. Examples include an ultrathin strain-gauge array (overall area of 10 mm^2^, 260 nm thick) based on 8 × 8 Si-NM elements, each connected to the electrodes of a Si-NM transistor in a multiplexed-addressable array design^71^. Si-NMs offer gauge factors (~150) that are much higher than those of conventional metal strain sensors (usually <10). This flexible microsystem can conform to biological targets such as a human fingertip, thereby providing measurement capabilities for strain mapping under different applied pressure forces, with a low hysteresis of ~1.0% and scanning speed of 100 kHz. Another example reports a haptic measurement system using stretchable, carbon-nanotube-based strain sensors, by introducing a self-locking effect in the Hertz mode (radius of probe tip as 500 µm). Integration of the miniaturized platforms with a fingertip model can enable rapid assessment of Young’s modulus of soft biomaterials by an individual simple touch, in a range from several kPa to over 1 MPa with robust adaptability to various applications^72^. Continued efforts focus on improvements in device characteristics, including conversion to an accurate evaluation of tissue moduli and optimized schemes for integration with biological targets.

As outlined in the cases above, these microsystem technologies are of particular interest for their flexible/stretchable mechanics and miniaturized dimensions compared to conventional technologies that involve large, applied forces through suction, traction, torsion, etc. The results include capabilities for precise measurements and continuous monitoring with stable mechanical interfaces even on highly curved, soft surfaces. These features suggest potential use in clinical practice and in at-home diagnostics, including those relevant to evaluations of patients with skin or other tissue disorders^41^.

### Planar microsystems using MEMS-based concepts and technologies

**Fig. 3:**
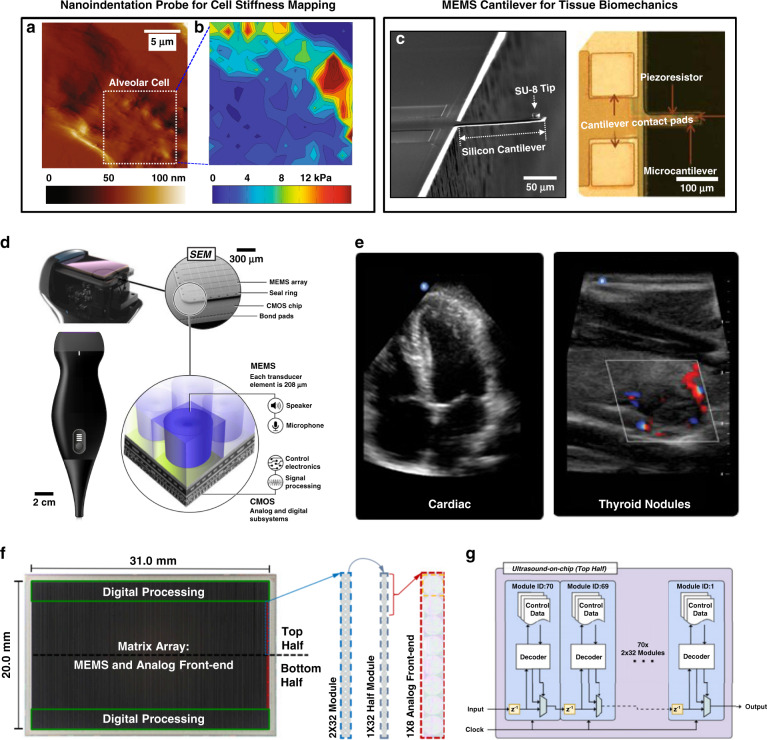
Planar microsystems using MEMS-based processing approaches. **a** Atomic force microscopy (AFM) image. A 10 × 10 µm^2^ area with a representative alveolar cell from a rat lung (dotted square) probed mechanically with 121 indentations. **b** Resultant AFM elastography mapping of the elastic modulus, rendered at an indentation depth of 300 nm. Reproduced with permission^73^. Copyright 2008, The American Physiological Society. **c** Scanning electron microscope (SEM) image of a piezoresistive microcantilever (left), consisting of a SU-8 indentation tip and doped silicon-ribbon cantilever as a piezoresistor, with a corresponding optical image of a microcantilever supported by a silicon wafer (right). Reproduced with permission^40^. Copyright 2014, Royal Society of Chemistry. **d** Ultrasound-on-chip (UoC) designs based on MEMS approaches. Photograph, SEM images, and schematic illustration of an ultrasound-on-chip design (left) that includes 8960 MEMS transducers (upper right) on a CMOS chip, each with control/processing circuitry for sending and receiving ultrasound signals (lower right). **e** Results of measured ultrasound images across cardiac and thyroid tissues, with color Doppler results showing small parts of the thyroid nodule. **f**, **g** Illustration of ultrasound-on-chip circuitry and chip integration. **f** A photograph of the electronic modules across the CMOS design. **g** Schematic illustration of the module-level communication architecture. Reproduced with permission^81^. Copyright 2021, National Academy of Sciences

Compared to the flexible/stretchable piezoelectric microsystems described in Fig. 2, rigid, planar microsystems based on MEMS approaches cannot conform to soft tissue surfaces but offer advantages in spatial resolution, functionality, and performance. Relevant examples of particular interest appear in this section, with features and capabilities that can complement those of devices outlined in the preceding section, as illustrated in Fig. 3 for the case of biomechanical evaluations of tissues/cells with noninvasive measurement interfaces to soft biological targets.

AFM elastography, which exploits nanoindentation approaches, provides for the spatial mapping of elastic moduli across two-dimensional intercellular surfaces in a “pointwise” manner^73,74^, as shown in Fig. 3a, b. Figure 3a features an AFM image of surface topography for a representative alveolar epithelial cell (3~5 µm thick) from a rat lung, which comprises cell elements such as nuclei and associated lamellar bodies. Here, standard pyramidal probes (silicon-nitride at nanoscale diameters) with known spring constants (~0.03 N/m)^75,76^ determine force-depth relationships at depths of hundreds of nanometers based on optical measurements of the tip position and electrical measurements of the displacement of the sample support. Figure 3b presents maps of elastic moduli defined in this manner across the perinuclear region (10 × 10 µm^2^, white square in Fig. 3a); 121 individual indentations, performed in a pointwise fashion, were executed in an 11 × 11 array of separate modulus measurements at a resolution of 1 µm^73^. The broad stiffness range of 1~15 kPa observed in the results includes peaks at locations of the lamellar bodies. Such data on intracellular mechanical heterogeneities can be valuable in research contexts, although the measurement mechanisms, including the optical readout approach, create barriers for routine use for in vivo monitoring of biomechanics in living animals and human subjects^77,78^.

By comparison to AFM techniques, Fig. 3c illustrates an example of a piezoresistive microcantilever structure (130 µm long, 40 µm wide, ~2 µm thick) built on a device-grade silicon wafer substrate for mechanical phenotyping of soft biological targets in a manner that bypasses the need for an optical readout mechanism^40^. Beyond standard AFM probes, customized probes can be designed for specific biological targets and measurement applications, where key parameters include constituent materials and corresponding geometries. The cantilever beam consists of a boron-doped silicon membrane (800 nm thick) that serves as a piezoresistive element (~500 Ω as undeformed configuration) connected by traces of Au (500 nm thick) with encapsulation layers of photolithographically patterned silicon-nitride. The microcantilever presents a spring constant of ~0.15 N/m, and the probe tip incorporates a cylinder structure formed in a photodefinable epoxy (10 µm diameter, 8 µm high; SU-8), as shown on the left side of Fig. 3c^79^. Pressing the tip onto the surface of a biological target leads to deformations of the microcantilever and associated changes in electrical resistance of the silicon piezoresistive element that depend on the target tissue biomechanics^80^. Ongoing efforts using this scheme focus on the use of mechanical properties to distinguish between healthy and diseased tissues, specifically cancerous tumors, and their high stiffness relative to those of healthy tissues.

Compared to these measurement approaches, active semiconductor-based MEMS devices that support signal amplification and multiplexed addressing, adapted from the integrated circuit industry, provide advantages in performance for evaluating deep-tissue biomechanics. As an example, Fig. 3d highlights the use of silicon technology as the basis of an ultrasound-on-chip (UoC) platform for medical imaging and analysis, where a dense array of 8690 MEMS transducers in a 140 × 64 configuration (area of 30 mm × 13.3 mm, spacing pitch of 200 µm) directly integrate with an underlying complementary metal–oxide–semiconductor (CMOS) electronic substrate^81^. Such ultrasound-on-chip platforms can be paired with a mobile device and artificial intelligence system to enable quantitative measurements with clinical-quality images of deep tissues across the human body, including the heart and thyroid (Fig. 3e).

Of particular interest, the underlying CMOS circuits (area of 31 mm × 20 mm) serve not only as front-end sensing electronics integrated with the transducer array and targeted tissues but also as a support for matrix addressing of each transducer to allow transmission of ultrasonic waveforms and detection of associated mechanical vibrations for back-end data acquisition, amplification, and processing (Fig. 3f, g). Specifically, Fig. 3f demonstrates the circuit strategies and chip integration schemes, where the 2 × 32-element ultrasound-processing-unit (UPU) modules distribute across the top and bottom halves of the matrix array, and each half-module (1 × 32 column) incorporates 4 analog front ends (red frame) that service 8 MEMS transducers. Figure 3g shows the digital communication subsystem between the 2 × 32-element UPU modules, serving as the input/output interfaces of each unit for signal recording and amplification. The overall circuit can individually drive each MEMS probe for transmitting ultrasonic waves and receiving vibrations, which are converted to electronic signals and thus processed, with beam-forming capabilities for steering and active focusing at a maximum depth of ~15 cm and with an imaging resolution of ~200 µm. The appeal of this platform is its potential as a robust diagnostic tool for automated monitoring of physiological parameters such as cardiac ejection fraction and associated tissue biomechanics.

Compared with the stretchable and flexible ultrasonic array described previously (Fig. 2e), which relies on piezoelectric materials diced into individual transducers, an important feature of these semiconductor-based devices with CMOS technology is that the active electronics themselves, consisting of high-density MEMS elements, many thousands of amplifiers, analog-to-digital converters and associated processing circuits, not just the transducers, integrate directly with soft tissues at impressive levels of functionality^43^. The results enable local signal amplification and high-efficiency data processing (1 trillion operations per second), with programmable measurement depths. On the other hand, the rigid, planar architectures of these systems may limit their utility compared to flexible, stretchable platforms when used on curved surfaces of the body and/or in a continuous wearable fashion.

**Fig. 4:**
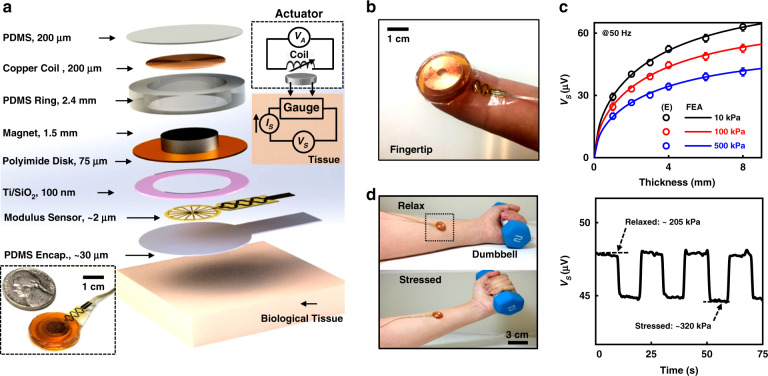
Miniaturized MEMS devices for the characterization of tissue biomechanics. **a** Exploded-view schematic illustration of the system. The upper inset shows a circuit diagram (upper-right). The lower inset is a photograph of the back side of the system. **b** Photograph of the device on the fingertip. **c** Experimental (E, symbols) and simulation (FEA, line) results of sensor voltages as a function of sample thicknesses with various moduli. **d** Dynamic measurements for modulus sensing on the human body. Left: Photographs of the forearm before (upper) and after (lower) lifting a dumbbell. Right: sensor voltages as a function of time during signal recording. Reproduced with permission^42^. Copyright 2021, Nature Publishing Group

Other MEMS-based approaches offer much simpler routes for measuring moduli. Figure 4a presents a schematic illustration of an electromechanical modulus (EMM) sensor that integrates a vibratory actuator operated at a fixed frequency to deliver periodic force to underlying tissue with a strain-gauge sheet for measuring the amplitude of the resulting displacements^42^. The force/amplitude relationships define the mechanical properties in a compact, disk-shaped platform with a thickness of 2.5 mm and an area of 0.5 cm^2^. Such devices can mount on a surface of interest, such as the skin, via gentle lamination in a reversible fashion that enables multiple cycles of use, as shown in an example on the fingertip in Fig. 4b. Figure 4c indicates that the voltage recorded by the strain gauge, *V*_*S*_, decreases with the increasing modulus (10 kPa to 500 kPa) and increases with increasing tissue thickness (up to ~8 mm) in measurements on phantom skin samples of poly(dimethylsiloxane) supported by glass wafers. Such characteristic depth enables effective measurements across a range of soft biological targets of the human body, including skin, subcutaneous fat, and muscle layers below the surface. As an example, the results of real-time monitoring of the effective mechanical properties of near-surface muscle tissue during activity appear in Fig. 4d. Here, the elastic modulus of the forearm is evaluated at a characteristic depth of ~8 mm in a relaxed state and a tense condition due to lifting a dumbbell. Continuous recording of *V*_*S*_ as a function of time during repetitive cycles of movements yields modulus values of 205 kPa and 320 kPa, corresponding to each state of muscle activity, with trends compatible with modulus measurements of muscles with variable contraction intensities via ultrasound elastography^82,83^. Furthermore, studies with human subjects demonstrate capabilities for precise evaluation of elastic modulus at various positions over body regions, including options in the use of arrays of such devices for large-area mapping of moduli.

As outlined in these MEMS-based approaches, an important feature is miniaturized dimensions that allow the application to biological targets ranging from cells to tissues and organs. Although promising, the planar, rigid architectures of some of these approaches (e.g., AFM techniques, microcantilevers) limit samples to cultured cells or tissue slices due to difficulties in application to curved surfaces or for use in monitoring time-dependent changes. Emerging microsystem approaches enabled by interdisciplinary advances in materials, manufacturing technologies, and engineering designs offer some compelling options in this context. Table 1 summarizes the microsystem approaches for all the cases outlined from Figs. 1–4, with key materials, fabrication techniques, and corresponding applications^31,40–45^. A key frontier area is in the development of flexible/stretchable materials for compliant platforms that not only conform to targeted tissues/organs but also offer a combination of advanced form factors and high-performance measurement functionality across measurement scales and depths, as outlined in various examples that follow.

**Table 1:** Summary of representative microsystems and their fabrication methods, with key materials, advantages, areas for improvement, and corresponding applications

System	Key materials	Main fabrication methods	Advantages	Room for improvements	Applications
AFM^73^	SiN_x_	Probe: focused ion beam, e-beam lithography, micromachining	Probe tips in high-resolution for cellular scale evaluation	Improving the scope of spatial scales and measurement stabilities for broader utilities of application targets	Cell stiffness evaluation
CBM^40^	Si/SU-8	Cantilever: spin coating, photolithography, chemical vapor deposition, reactive ion etching (RIE)	Fabrication is designed with unique form factors in terms of applications	Reducing measurement uncertainties, particularly on curved, dynamics surfaces	Malignant tissue characterization
PAT^41^	PZT films	Actuator/sensor: spin coating, sol-gel, photolithography, transfer printing, e-beam deposition, Wet Etching, RIE, laser cut	Flexible platforms in large arrays for the conformal contract during the measurement	Improving penetration depths by optimizing the designs of PAT microsystems	Biomechanical evaluation of the superficial region
OCE^31^	Optical materials	Light emitters: spin coating, photolithography, micromachining, mold casting	Measurements via photophysical interactions non-invasively	Reducing the light attenuation and improving penetration depths via integrating additional optical waveguide	Stiffness mapping via optical illumination
EMM^42^	Au/PDMS	Strain gauge: spin coating, photolithography, transfer printing, e-beam deposition	Characterization of soft tissue mechanics at tunable depths	Further miniaturizing the design dimensions and integrating active materials to enhance measurement capabilities	Depth profiling of biomechanics of skin, fat, and muscles
US^43^	PZT dices/piezoelectric materials	Transducer: dicing and filling, micromachining, mold casting, laser ablation	Deep-tissue mapping based on ultrasonic device array in a stretchable format	Improving reliability on irregular surfaces and integrating acoustic metamaterials in wearable design	Deep-tissue sensing for physical structure properties

### Biomechanical evaluations across various measurement depths

**Fig. 5:**
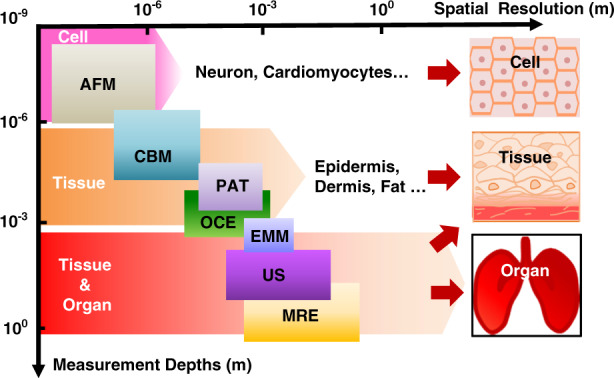
Summary of microsystem technologies with various measurement scales. Illustration of the approximate ranges of measurement depths and spatial resolutions for various biological targets via different types of evaluation methods, including AFM, cantilever-based measurement (CBM), piezoelectric-actuator/sensor techniques (PAT), optical coherence elastography (OCE), electromechanical modulus (EMM) sensors, ultrasound techniques (US) and magnetic resonance elastography (MRE)

Measurement scale, such as sensing depth, is an important parameter associated with each of these methods and is of essential relevance to assessments of depth-dependent structures commonly found in biological tissues^1^. Figure 5 summarizes the literature results for measurement depths (from 10^−9^ to 10^0^ m), along with spatial resolutions that correspond to various evaluation approaches and biological targets such as cells, tissues, and large organs at nanoscale, microscale, and macroscale dimensions^8,84^. The differences in mechanics measured at different scales depend on the structures of the tissues themselves; these differences are addressable using different measurement modalities and device designs.

AFM elastography methods generally use a standard probe (e.g., typically as silicon-nitride on the nanometer scale) for the shallowest measurement depths (<10^−6^ m), with the highest spatial resolution (which refers to the diameter of the probe as 10^−9^~10^−6^ m)^85^; this method is suitable for evaluating individual cells and substructures via nanoindentation at a temporal resolution of ~10^1^ Hz^86^. Elastic modulus evaluations generally rely on measurements of the relationship between nanoindentation forces and probe displacements, the results of which can evaluate cell biology at nanoscale dimensions. Current efforts focus on the use of such methods for monitoring cell physiology (i.e., growth and repair) and evaluating abnormal variations in cell stiffness associated with pathological conditions. For example, in prior work, the mechanical nanoindentation of breast tumor tissues associated with cancer cells in human subjects was investigated, with correlative stiffness maps of both normal and benign regions^87^. Associated measurement modes involve probe tips with a resolution of 40 nm to measure the relationship between force and displacement across tissue areas of 20 × 20 µm^2^ at nanoscale penetration depths (>150 nm).

Custom piezoresistive microcantilevers support enhanced measurement scales and capabilities that can complement those of standard AFM approaches. The designs can be tailored to biological targets of interest through optimized choices of materials and geometries. Custom-built cantilever-based (CBM) techniques can also support sensing depths at larger scales (10^−6^~10^−5^ m)^45^, mostly corresponding to lateral resolutions ranging from 10^−7^ to 10^−5^ m and temporal resolutions from 10^0^~10^2^ Hz^40^. The results can enlarge the scope of measurement targets (e.g., tissues, cells) and spatial scales (from nanoscale to microscale dimensions). Examples include mechanical measurements of a single-neuron of a rat model in an ex vivo test manner that includes cultured neuronal cells on a foreign substrate. Measurements use a silicon-nitride probe (area of 50 × 30 µm^2^, ~1 µm thick) at a single-neuron resolution. Indentations on the cell surfaces can yield neuron modulus evaluations with an increase of ~0.7 kPa after treatment with a nerve growth factor at penetration depths at the micrometer level^45^.

The thin, flexible microsystems using PAT as described previously (i.e., filamentary probe in Fig. 2c, conformal sheets in Fig. 2d, etc.) can yield tissue moduli for near-surface regions of biological targets over characteristic depths of 10^−5^~10^−4^ m^30,88,89^. Of note is their ability to support two-dimensional arrays distributed across macroscopic areas, with a lateral resolution from 10^−4^ to 10^−3^ m and temporal resolution of <10^3^ Hz. An example is shown in Fig. 2d, where the microsystems are capable of measuring the mechanics of near-surface regions of tissues. The thin, conformal structure allows direct lamination on human skin for measurements with a spatial resolution of 500 µm. The penetration depths are restricted to the submillimeter range^41^.

Compared to the methods outlined above, OCE techniques can yield insight into deep-tissue mechanical responses under externally applied forces. For example, tissue stiffness characterization can be used for quantitative mapping of elastic moduli across benign and malignant breast tissues at a measurement spatial resolution in the submillimeter range, a temporal resolution of 10–10^4^ Hz, and penetration depths over 1 mm^30,31^. The results indicate an elasticity of ~410 kPa for malignant regions, which is two orders of magnitude higher than that of benign regions. Extensions of this method based on the use of optical waveguides and optimized selection of the illumination wavelength can enhance the penetration depth^30^. Beyond these schemes, EMM devices (Fig. 4) can perform in vivo mechanical sensing at characteristic depths ranging from 10^−3^ to 10^−2^ m, with a lateral resolution from 10^−3^ to 10^−2^ m and a temporal resolution of ~50 Hz, depending on the designs. For example, such devices enable the evaluation of soft tissue biomechanics not only in the near-surface, superficial regions (e.g., epidermis, dermis) but also for deep structures of bulk targets (e.g., fat, muscles) at tunable depths ranging from 1 to 8 mm depending on the particular device design^42^.

For profiling the mechanical properties of soft tissues beyond such depths, ultrasound imaging exploits reflections of traveling waves at both tissue and organ scales, with measurement depths of 10^−1^ m, a spatial resolution of 10^−4^~10^−3^ m, and a temporal resolution of ~10^2^ Hz^30^, limited mainly by acoustic attenuation. Related examples displayed in Fig. 3d associated with the UoC system can support matrix addressing to allow the transmission of ultrasonic waveforms and mechanical detection. Another example is the MRE method, which provides a spatial-temporal mapping of both tissue and organ stiffness for applications such as localized identifications of fibrotic tissue in the liver^90^, with a large characteristic depth (10^−3^~10^0^ m) and a spatial/temporal resolution of ~10^−3^ m/~10^2^ Hz^91,92^. Future efforts for all of these technologies are in the development of microsystem approaches that combine the capabilities of mechanical evaluation without constraints associated with measurement scales and depths from superficial cellular regions to deep, large organ geometries.

### Clinical applications of mechanical sensing/monitoring

**Fig. 6:**
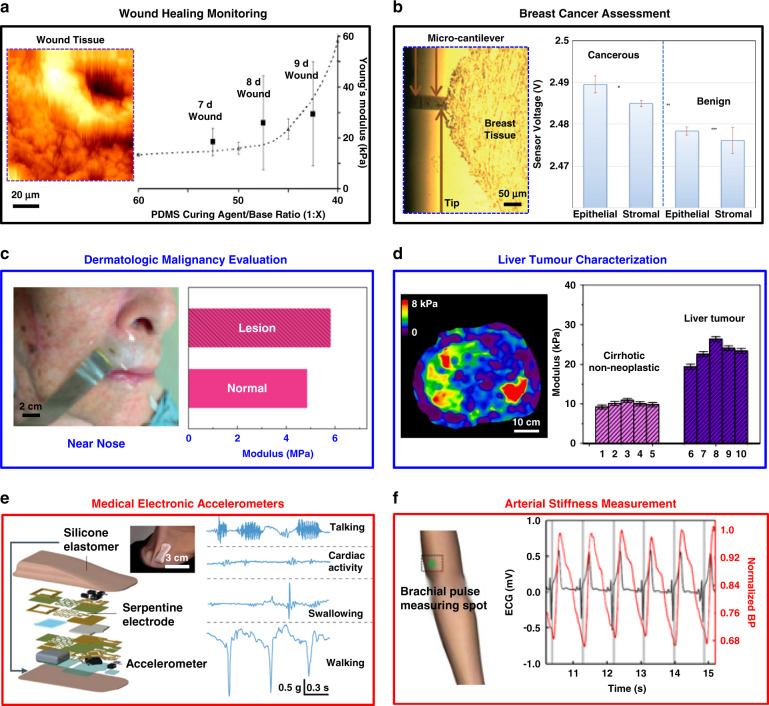
Clinical applications of mechanical sensing/monitoring. **a** Left: Topography image of a 100 × 100 µm^2^ area of a section of 9-d-old granulation tissue in a rat model. Right: the mean elastic moduli determined via the AFM method applied to granulation wound tissue from a rat after 7, 8, and 9 days as a function of different weight ratios of the base to crosslinker in PDMS with comparable moduli (right). The error bars correspond to standard deviations for measurements among 12 rats. Reproduced with permission^92^. Copyright 2006, Rockefeller University Press. **b** Left: microcantilever indenting a sample of breast tissue associated with breast cancer. Right: sensor voltages during measurements on cancer epithelial/stromal tissues and benign epithelial/stromal tissues. Reproduced with permission^40^. Copyright 2014, Royal Society of Chemistry. **c** Photographs of a piezoelectric device mounted near the nose to map the elastic modulus in lesion regions associated with dermatologic malignancy. Reproduced with permission^41^. Copyright 2015, Nature Publishing Group. **d** Left: magnetic resonance electrographs of a cirrhotic explanted human liver with a tumor. Right: modulus values measured from healthy and cancerous tissues using needle-shaped PZT-based probes^65^. Copyright 2018, Nature Publishing Group. **e** Microsystem approaches for MA measurements. Left: Schematic illustration of skin-mounted electronics for MA sensing, with an inset that shows an image of a device on a human subject. Right: Continuous MA-signal recording of human body orientations. Reproduced with permission^94^. Copyright 2020, Nature Publishing Group. **f** Left: schematic illustration of measurements of brachial arterial-pulse via ultrasonic devices. Right: recording of BP waveforms correlated with ECG results, in which the pulse wave velocity can be calculated as 5.4 m/s. Reproduced with permission^43^. Copyright 2018, Nature Publishing Group

Promising clinical opportunities are in biomechanical evaluation at nanoscale, microscale, and macroscale dimensions (Fig. 6). Here, examples include (a) tracking of wound mechanics via nanoindentation in rat models during healing^93^, (b) mechanical assessments of breast tissues for cancer diagnostics^40^, (c) evaluation of human skin lesions with dermatologic malignancy^41^, (d) characterization of the human liver tumors via biopsy needles^65^, (e) electronic accelerometers for mechano-acoustic sensing^94^ and (f) measurements for human arterial stiffness^43^.

Figure 6a presents an example that involves monitoring wound biomechanics associated with healing processes^93^. Here, AFM elastography captures the Young’s modulus of wound granulation tissues in rat models (wound area of 2 × 2 cm^2^, at full skin thickness) during spontaneous healing. The experiments involve dissecting tissue samples at different timescales and sectioning them into slices (150 µm thick) for characterization^95,96^. The results reveal a significant increase in the wound modulus from ~19 kPa to ~29 kPa during healing (from 7 days to 9 days), primarily due to an increase in the density of fibrotic tissue in the wound bed. The value continuously increases to a maximum of ~49 kPa as the wound almost closes after 12 days. Another similar example features nanoindentation measurements on slices of human pituitary gland tissue (~1 mm^2^, 150 µm thick). The experiments include 1285 indentations as the basis of spatial maps of elastic moduli across an area of 95 × 95 µm^2^ on the tissue slice^74^. The results indicate Young’s modulus values that span a wide range from ~1 kPa to ~50 kPa, with spatial gradients that reach 12 kPa/µm, demonstrating the mechanical heterogeneity of human brain tissues.

Related microscale measurements via MEMS-based cantilever structures can enable mechanical phenotyping of soft biological targets (Fig. 3c). Figure 6b presents a case of the clinical use of mechanical measurements to distinguish benign and cancerous breast tissues. Tissue microarray (TMA) technologies produce ultrathin tissue slices from breast cancer patients (0.6 mm diameter, ~4 µm thick) for mechanical analysis via the piezoresistive sensor system^40^. The results from epithelial and stromal regions throughout benign and cancerous regions (right of Fig. 6b) indicate variations in mechanical properties that can differentiate benign from malignant tumors^95^.

Figure 6c shows the clinical utility of wearable, conformal PZT arrays of actuators/sensors (~20 µm thick) for identifying and targeting dermatological lesions using the platform in Fig. 2d. Example measurements near the nose of a subject with skin malignancy reveal that the modulus of the lesion (~6 MPa) is higher than that of healthy regions (~4.5 MPa), consistent with cancerous tissues that are typically stiffer than healthy tissues^97,98^. This mode of operation provides utility in clinical investigations of the progression of skin tumors and in the assessment of the metastatic potential of cancer cells^41^. Another example is in clinical use of the electromechanical device described in Fig. 4^42^. Quantitative assessments across lesioned and nearby healthy regions of the skin of patients with psoriasis highlight a representative application in dermatology. Measurements on the psoriasis region of the patient indicate an increase of ~120 kPa in modulus compared to that of nearby skin due to the pathological structural changes related to bulk skin properties such as thickness, stiffness and hydration. These simple measurements establish quantitative metrics that can serve as diagnostic markers for a wide range of skin conditions, suggesting clinical significance for the monitoring and diagnosis of relevant health disorders^39^.

In addition to skin disorders, the thin platform of the needle-shaped probe (as described previously in Fig. 1c) provides powerful capabilities for the diagnosis of internal tissues, as shown in Fig. 6d^65^, with a specific demonstration in the context of hepatocellular carcinoma (HCC). The unique geometry of the probe enables measurement with guided biopsy needles for HCC in a small tumor region within the liver (<3 cm). Associated measurements of human livers with cancerous lesions reveal higher elastic modulus values (~25 kPa) than those (<10 kPa) obtained from healthy nonneoplastic tissues nearby (right in Fig. 6d). These results are consistent with those based on MRE (left in Fig. 6d).

Beyond tissue modulus measurements, advanced microsystems, which serve as the basis of electronic stethoscopes and accelerometers for mechanical vibration sensing, can also yield information on biomechanical–related properties of biological targets, including mechano-acoustic (MA) signals^94^. Recent studies have established a soft, skin-mounted electronic device designed to conform to the human suprasternal notch (Fig. 6e) with wireless operation. The MA signals span a wide range from subtle vibrations at accelerations of 10^−3^ m/s^2^ to large body motions of 10 m/s^2^ at frequencies up to 800 Hz. The results yield measurements of physiological processes and body motions, such as talking, cardiac activity, swallowing, and walking, corresponding to various vibration frequencies. Ongoing work focuses on incorporating additional multiple mechanical sensors for signal processing and precise measurement of tissue mechanics.

Another example in Fig. 5f involves ultrasonic devices for the assessment of human arterial stiffness^43^. Similar to the construction described in Fig. 2e, this stretchable microsystem consists of a 4 × 5 array of piezoelectric transducers (spatial resolution of 900 µm, 240 µm thick) designed to capture blood pressure (BP) waveforms at deeply embedded arteries and veins connected to cardiac tissues. This wearable platform can establish conformal contact with curved skin surfaces to provide deep-tissue monitoring, such as central BP of deep vasculature. The results are consistent with those of a commercial tonometer. Figure 6f (right) shows BP waveforms tested at the brachial artery (red), correlated with simultaneous recordings of electrocardiogram (ECG) data (black), where the arterial-pulse-wave velocity can be measured (5.4 m/s, proportional to artery modulus) to evaluate arterial stiffness, as a key determinant of cardiovascular risk. Such noninvasive microsystem technologies offer accurate monitoring of the mechanical properties of deep biological organs and create opportunities for diagnosing a broad range of related cardiac diseases. Further efforts include implementing phased-array control algorithms to enable focusing/steering of the ultrasonic beam^66^.

### Challenges and future directions

The results highlighted in this review represent some of the most recent microsystem technologies deployed in animal models and human subjects for biomechanical sensing. Progress in materials science, device engineering, and measurement principles establish the foundations for a collection of high-precision measurement systems as tissue-compatible platforms with advanced capabilities for the mechanical characterization of soft biological tissues. These types of approaches provide key features beyond those supported by conventional methods, with unique modes for interfacing with soft tissues, enhanced measurement resolution, and an expanded scope of applications. The resulting capabilities offer future opportunities in diagnostic utility in terms of different types of disorders that range from characterizing microscale fibrous histiocytoma to performing large-area dermatologic imaging and identifying deep tumors below the surface. Although many of the examples outlined here are appealing, a persistent challenge is in the development of a single, scalable measurement system with hybrid functions that yield evaluations of biomechanics at a combined set of spatial scales and depths, all in a rapid, precise in vivo manner without invasiveness.

Additional areas for future work are in improving the performance of these microsystem approaches, where increasing the measurement accuracy and precision and the spatial resolution represent key aspects. Continued efforts are in optimization of material engineering, manufacturing technologies, assembly of high-density sensor/actuator arrays, and further miniaturized system designs. In mechanical sensing, replacing strain gauges based on conventional metals (e.g., Au and Pt) with semiconductors such as single-crystalline silicon nanomembranes or with piezoelectric materials such as PZT could lead to enhancements^99,100^. For clinical diagnostics, simplicity in designs and operation could lead to routine tools for precise, high-sensitivity biomechanical evaluations^101^ as alternatives to qualitative examinations based on manual palpations.

An important trend for many of these technologies is the integration of functional microsystems into wearable or portable platforms with wireless operation and interfaces with smartphones for routine measurements and at-home diagnostics. An ultimate goal is in real-time, continuous monitoring of patients on the basis of tissue biomechanics, as a complement to traditional vital signs, for tracking pathophysiologic changes or providing feedback to therapy. Designs that combine actuating/sensing components with interfaces to advanced data analytics approaches, all in battery-free platforms that can be worn on the body, are particularly attractive. Ongoing interdisciplinary efforts in this direction focus on constituent materials, mechanics designs, integration schemes, and measurement performance. These and other efforts based on the technologies summarized in this article will accelerate further progress and thus create future opportunities for exploratory research as well as for eventual clinical translation.
